# Deep Learning-Based Prediction and Compensation of Performance Degradation in Flexible Sensors

**DOI:** 10.3390/mi17040496

**Published:** 2026-04-18

**Authors:** Zhiyuan Wang, Tong Zhang, Luyang Zhang, Xiao Wang, Youli Yao, Qiang Liu, Yijian Liu, Da Chen

**Affiliations:** College of Electronic and Information Engineering, Shandong University of Science and Technology, Qingdao 266590, China; wangzhiyuan@sdust.edu.cn (Z.W.); zhangluyang@sdust.edu.cn (L.Z.); wangxiao@sdust.edu.cn (X.W.); ylyao@sdust.edu.cn (Y.Y.); qiangliu@sdust.edu.cn (Q.L.); liuyijian@sdust.edu.cn (Y.L.)

**Keywords:** flexible CNT deformation sensors, performance degradation prediction, GAN data augmentation, sequence attention transformer, smart glove

## Abstract

Flexible deformation sensors inevitably suffer from sensitivity degradation and severe measurement errors during long-term cyclic stretching due to structural fatigue. Traditional material-level optimizations are costly and lack dynamic adaptability. Herein, we propose an artificial intelligence (AI)-driven predict-and-compensate framework for the online calibration of flexible sensors. To overcome training sample scarcity, a generative adversarial network (GAN) performs temporal data augmentation. Subsequently, a hybrid deep learning framework integrating long short-term memory (LSTM) networks and a Sequence Attention mechanism is employed. This architecture accurately captures both local signal fluctuations and multiscale long-term decay trends, enabling precise multi-step prediction and output compensation. Experimental evaluations validate that this strategy significantly suppresses sensor response drift. Under cyclic loading, an initially substantial relative measurement error of 48.63% plummets to 7.16% post-calibration, with typical errors consistently reduced to the ~1% level. Furthermore, when deployed in a smart glove gesture recognition system, this method successfully restores the recognition accuracy from a fatigue-induced low of 75.73% (after 200 stretch cycles) back to 97.70%. This generative and attention-based deep learning paradigm offers robust, real-time error calibration, providing a highly viable solution for extending the long-term reliability and stability of flexible sensor systems.

## 1. Introduction

Flexible deformation sensors have become core technologies for health monitoring, human–machine interaction, and wearable electronics [1,2,3,4,5]. Based on their transduction mechanisms, these devices are predominantly categorized into capacitive and resistive sensors. Capacitive sensors typically exhibit excellent long-term stability and minimal baseline drift [6,7], but their application is sometimes constrained by complex fabrication and relatively lower sensitivity. In contrast, resistive strain sensors are widely favored for their exceptionally high sensitivity [8,9] and simpler read-out circuitry.

However, for long-term applications, measurement stability and repeatability are far more critical than instantaneous sensitivity [10,11,12]. Unfortunately, long-term cyclic operation inevitably induces baseline drift, significant hysteresis, and sensitivity loss. This degradation is a universal bottleneck faced by nearly all resistive strain sensors—whether based on silver nanoparticles, carbon nanoparticles, silver nanowires, graphene, liquid metals, or carbon nanotubes [7,13,14,15,16,17]. Given the universality of this challenge, developing a robust, dynamic compensation framework holds broad significance for the entire field of resistive flexible electronics. In this study, carbon nanotube (CNT) sensors are selected as a representative case. Their operation relies on the strain-responsive nature of the CNT network [18,19,20]. During prolonged cyclic deformation, the conductive pathways degrade due to nanotube slippage, microcrack propagation, and matrix viscoelastic relaxation [20,21]. Consequently, continuous stretching produces cumulative measurement errors, with some devices exhibiting irreversible resistance drift exceeding 30%, severe hysteresis, or even complete failure under severe fatigue [10,22,23,24]. Such performance degradation is often exacerbated by microstructured designs—such as microcracks, wrinkles, or pyramids—which are typically employed to enhance sensitivity at the cost of accelerated cyclic fatigue [11,25,26,27,28,29,30]. To mitigate these issues, previous efforts have explored traditional material-level optimizations, including tailored composite formulations and electromagnetic patterning [18,23,31,32,33,34,35,36]. Nonetheless, these approaches generally increase process complexity, entail prohibitive time costs, and often sacrifice flexibility [21]. Given the presence of irreversible structural damage, material-only solutions are unlikely to fully resolve the degradation issue, necessitating the introduction of advanced signal processing strategies.

Considering that most flexible CNT deformation sensors exhibit pronounced nonlinear responses, artificial intelligence algorithms are well-suited for their signal processing [37]. While deep learning has been widely successful in perception-level tasks for flexible sensors (e.g., gesture and motion recognition [38,39,40,41,42,43,44,45,46,47]), and generative models have been utilized to expand sample spaces and improve generalization [48,49,50,51,52,53], direct application of deep learning to device-level degradation prediction and online compensation remains relatively rare. However, deep-learning–based damage prediction and remaining-useful-life estimation in areas such as material fatigue [52,53,54], crack propagation [55], and sensor drift [56,57,58,59,60] offer valuable precedents. Prior work shows that by learning mappings between damage states and performance metrics, neural models can predict material property degradation, such as loss of stiffness [53], reduction in elastic modulus [52], or changes in electrical properties [61,62]. Furthermore, generative time series models have been used to augment scarce samples of severe degradation [51], while physics-informed frameworks inject classical mechanics priors to enhance credibility [63]. At the same time, fusing neural outputs with online estimation methods enables real-time calibration and compensation [64,65]. Collectively, these studies indicate that deep algorithms can learn and forecast macroscopic degradation driven by fatigue and cracking, offering a feasible methodological foundation for predicting and compensating attenuation in flexible CNT deformation sensors.

Hybrid time series modeling that combines LSTM and Transformer architectures has recently drawn attention in engineering prognostics, for example, in battery remaining useful life prediction [66,67], tire wear estimation [68], machinery fault diagnosis [69], and rocket ignition monitoring [70]. Particularly, advanced Transformer variants have demonstrated exceptional capabilities in tracking performance degradation and estimating the remaining useful life of complex mechanical systems [71,72]. LSTM models are well-suited to capture short-term and local dynamic features [73,74], whereas Transformer variants excel at modeling global, long-range dependencies and enable parallel training [75,76]. Integrating the two architectures achieves a complementary local–global decomposition that enhances cross-segment information fusion and improves long-sequence modeling performance. However, two core practical challenges arise in real applications. First is the small-sample problem: a single sensor typically provides only a limited number of degradation cycles, which is insufficient to support the training and generalization of complex models. Second, the strong periodicity of the signals produces many highly similar segments, which can mask the subtle degradation signatures associated with microstructural evolution, thereby making it difficult for models to reliably detect these features.

**Figure 1:**
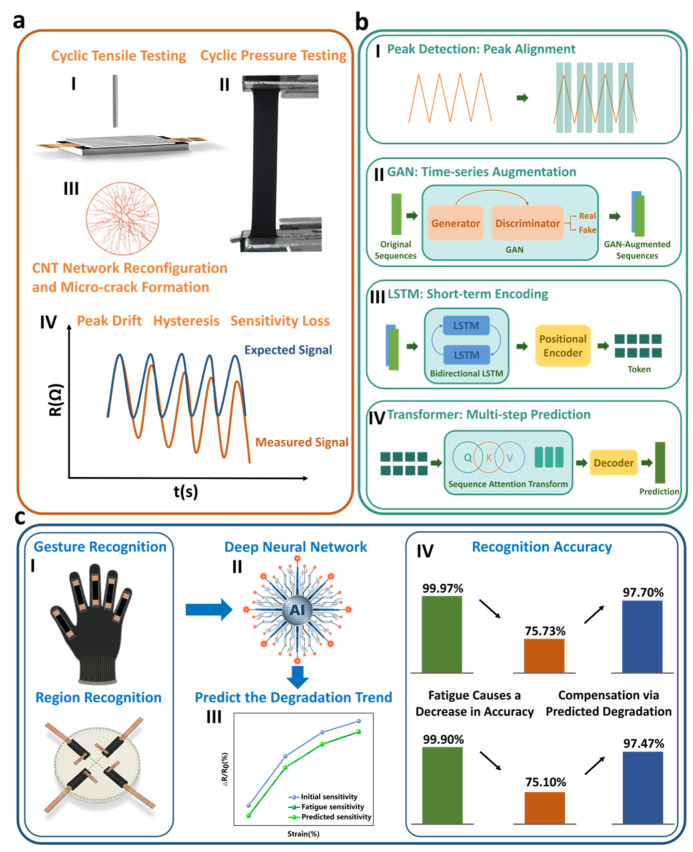
CNT sensors and the proposed solution. (**a**) CNT sensor performance degradation: (I) pressure cycling test, (II) tensile cycling test with deformation, (III) CNT network reorganization and microcrack initiation, (IV) macroscopic performance degradation phenomena in CNT sensors. (**b**) LSTM multiscale sequence attention Transformer predictive model: (I) peak detection mechanism, (II) GAN, (III) LSTM, (IV) sequence attention-based Transformer. (**c**) Prediction results and compensation application: (I) the predict–compensate mechanism applied to gesture recognition and region recognition, (II) neural network prediction of the degradation trend of sensor performance, (III) predicted degradation trajectory, (IV) compensation that largely eliminates recognition accuracy loss caused by sensor fatigue.

Building on this momentum, to address in-use performance degradation of flexible CNT deformation sensors, we propose a deep-learning-based predict-and-compensate framework (Figure 1a) that forecasts device degradation and performs online measurement correction. To mitigate small-sample limitations, a generative adversarial network is employed for time-series augmentation, enabling the model to access a wider range of degraded trajectories, while a sequence-level attention mechanism replaces conventional pointwise self-attention to better capture cross-segment similarity in strongly periodic signals (Figure 1b). To validate the approach, we fabricated representative CNT deformation sensors, collected cyclic tensile data within a small strain regime, and trained the model on datasets exhibiting clear degradation trends. This study focuses on this low-strain regime (e.g., 5–20%), which is highly representative of subtle human–machine interaction and regional strain mapping. After compensation, the relative error under each strain condition was reduced to approximately 1%. We further integrated the framework into gesture-recognition and region-recognition systems (Figure 1c), and comparative experiments spanning initial to advanced fatigue states—including realistic idle–load cycling—show that the compensation mechanism substantially restores performance degraded by sensor fatigue, recovering accuracy close to the original baseline. These results indicate that the proposed method can extend the usable lifetime of flexible CNT deformation sensors and offers a practical route to improving long-term reliability in wearable human–machine interaction and rehabilitation-monitoring applications.

## 2. Materials and Methods

### 2.1. Cyclic Stretch Signal Acquisition and Preprocessing

The fabricated flexible sensor was clamped at both ends on a tensile tester and stretched at a velocity of 200 mm/min. The maximum stretch amplitudes were set to 5%, 10%, 15% and 20% of the sensor’s original length. Resistance was recorded with a digital multimeter (DM3086) at a sampling frequency of 10 Hz. During preprocessing, the raw signal was low-pass filtered to remove high-frequency noise and ensure signal quality, and then normalized. The normalization is defined as:

(1) $$\dot{x_{i}}=\frac{x_{i}-x_{0}}{x_{0}}$$

where $x_{0}$ is the resistance measured before stretching. Because resistance oscillations are large in the initial cycling stage and do not reflect the steady degradation trend, sampling begins after 700 stretch cycles; the subsequent 400 cycles are used as the experimental dataset and the final 200 cycles of those 400 are reserved as the target data for prediction (Figure S13). The first 200 cycles of this window are split into training, validation, and test sets in the ratio 0.75:0.15:0.10 in strict chronological order to prevent any temporal data leakage. It is crucial to note that to maintain a rigorous evaluation, the GAN-based time-series augmentation was exclusively applied to the training set. The validation and test sets consisted solely of unaugmented, real measurement data, ensuring an unbiased assessment of the model’s true predictive and generalization capabilities.

### 2.2. LSTM Multiscale Sequence Attention Transformer Model

The model first projects the preprocessed resistance sequence into the representation space required by the LSTM via an embedding layer. A two-layer bidirectional LSTM with 256 hidden units per direction encodes the time series in each sliding window, so that the LSTM output at each time step is a 512-dimensional vector after concatenating the forward and backward hidden states. To reduce the computational cost of the subsequent Transformer and to form segment tokens, segment-level aggregation is applied to several time-step hidden states within each subsequence; the aggregation window length is 64 with a stride of 32. Each aggregated 512-dim vector is linearly projected to a Transformer embedding dimension of 256 and augmented with learnable positional encodings to produce the token sequence. The token sequence first passes through a multiscale sequence attention module to fuse local and midterm dependencies and is then processed by a Transformer encoder to model cross-segment and long-range relationships; the Transformer consists of three encoder layers with a feed-forward dimension of 1024 and a dropout rate of 0.2 to mitigate overfitting. The Transformer output is fed to a trend-aware multilayer perceptron and finally decoded by the prediction head into multi-step degradation forecasts. Training uses the AdamW optimizer with an initial learning rate of 1.5 × 10^−5^, a minibatch size of 32 and up to 200 epochs. The loss is primarily mean squared error supplemented by extrema- and phase-aware penalty terms to enhance sensitivity and robustness to peaks, troughs and phase information. Crucially, when applied to the GAN training process, these physically inspired penalty terms prevent the generation of mathematically valid but physically distorted biases, ensuring that the synthetic samples faithfully replicate the true degradation and hysteresis patterns of the CNT conductive network.

### 2.3. Signals Acquisition and Processing for Gesture Recognition and Region Recognition

In both application scenarios, data were acquired with an EV80B Bluetooth acquisition board (EV80B, Xinyuan Electronics Studio, Zhengzhou, China) and the sampling frequency was fixed at 500 Hz. After the acquisition, the resistance signals output by the sensing unit were converted to voltage signals and transmitted to the host computer via the Bluetooth module and then filtered to remove noise. For gesture recognition, the dataset contains three gesture classes: scissors, rock and paper. Each class contains 10 samples and each sample records 2 s of signal, corresponding to 3 × 1000 data points. For direction recognition, the dataset contains four regions, each region contains 10 samples and each sample records 1 s of signal, corresponding to 4 × 500 data points. The above datasets are used only for training and testing. In addition, one sample from the initial condition, one sample after 100 fatigue cycles with compensation and one sample after 200 fatigue cycles with compensation were selected as the validation set to compare and verify the effectiveness of the compensation model.

### 2.4. MLP Classification Model

The classifier is implemented as a multilayer perceptron and the input dimension matches the normalized feature vector. The network comprises three parts. The first part is a fully connected layer with 128 hidden units and ReLU activation, followed by dropout with probability 0.4 to suppress overfitting. The second part is a fully connected layer with 64 hidden units and ReLU activation followed by dropout with probability 0.3 for regularization. The output layer is a fully connected projection followed by SoftMax to obtain class probability distributions. Training uses the Adam optimizer with an initial learning rate of 1 × 10^−4^. Training is performed in mini-batches with a batch size of 32 and a maximum of 200 epochs. To ensure generalization and support model selection validation accuracy, the validation set is used as the monitoring metric. ModelCheckpoint is used to save weights that achieve the highest validation accuracy and EarlyStopping is enabled to restore the best weights and terminate training if validation accuracy does not improve for 15 consecutive epochs. Data preprocessing uses min–max normalization computed from the training set column vectors and the normalization parameters are saved for reproducibility and deployment. After training, the best model is loaded and evaluated on the test set to report test loss and accuracy and to compute the confusion matrix and classification report for detailed performance analysis. Training loss and accuracy curves are also saved for convergence assessment and overfitting diagnosis.

### 2.5. Development Environment

The proposed prediction model was developed on a platform running Python 3.7.6 and PyTorch 1.13.0 with an Intel(R) Core (TM) i9-10900 2.8 GHz processor and 25.9 GB of memory.

## 3. Results

### 3.1. Fabrication and Characterization of Flexible CNT Deformation Sensors

**Figure 2:**
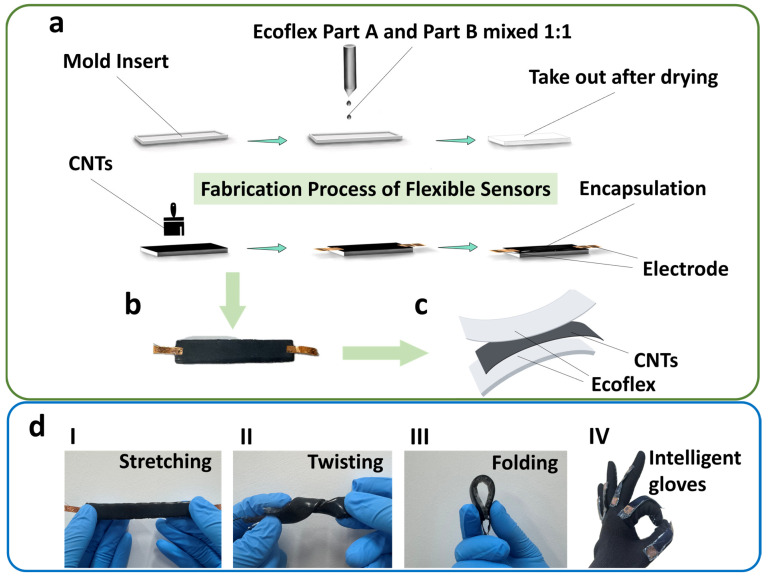
Fabrication and characterization of the flexible CNT deformation sensor. (**a**) The preparation process diagram of the sensor. (**b**) Photograph of the sensor. (**c**) Schematic diagram of the sensor structure. (**d**) Images of the sensor under bending, stretch deformation, twisting and in a smart glove, (I) stretching, (II) twisting, (III) folding, and (IV) application in intelligent gloves.

Deformation sensors that use CNTs as the conductive phase commonly exhibit performance degradation under cyclic loading. Among the frequently reported fabrication routes are brush-coating CNT powder onto the substrate, directly depositing CNT powder, and spray-coating a uniform CNT dispersion. To investigate degradation behavior, we fabricated and tested representative devices of all three types, and the brush-coating process is described below as an example. Ecoflex was chosen as the substrate because its cured surface remains adhesive and it provides the flexibility and stretchability required by the sensor. Owing to the fine particle size and excellent conductivity of CNT powder, the powder was applied onto the Ecoflex surface with a brush. Triboelectric effects and friction between the brush and the substrate cause MWCNT powder to adhere to and embed within the Ecoflex surface, thereby forming a conductive layer. The complete fabrication process (Figure 2a) involved pouring premixed and degassed Ecoflex (A:B) into a mold for thermal curing, followed by the brushing of a multi-walled carbon nanotube (MWCNT) layer, the application of electrodes using conductive graphene paste, and a final Ecoflex encapsulation. After final curing, the flexible sensor was ready for experiments. Photographs of the fabricated sensor and its structure are shown in Figure 2b–d presents images of the sensor under bending, stretching, twisting and integrated into a smart glove.

**Figure 3:**
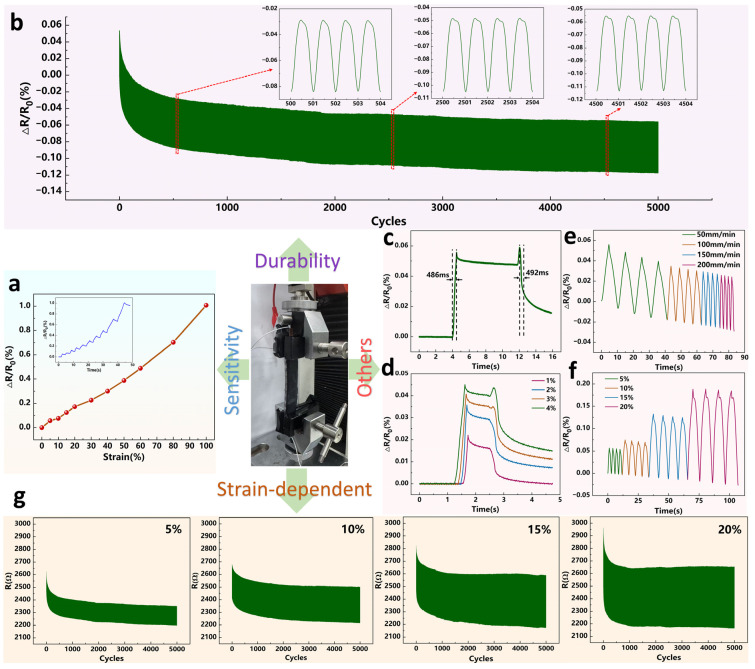
Fundamental tensile performance and data collection of the brush-coated CNT sensor. (**a**) Sensitivity curves of the sensor at 0–100% strain. (**b**) Durability test of the sensor. (**c**) Response time and recovery time at 5% strain of the sensors. (**d**) Comparison of response times under different small strains. (**e**) Cyclic resistance response of the sensors at 5% strain with different stretching rates. (**f**) Step response at various strain levels. (**g**) Collect the changes in the raw values of the cyclic tensile resistance of the sensor.

Tests showed that all three device types fabricated by different CNT loading methods exhibited varying degrees of degradation, and to ensure basic functionality, we performed detailed tensile characterization on the brush-coated CNT sensor. Figure 3a presents the sensor sensitivity curve over a strain range of 0–100%, showing stable operation across the entire test window without abrupt jumps or abnormal fluctuations. As shown in Figure 3b, the device underwent a durability test of 5000 continuous stretch-release cycles at 5% strain, revealing a clear attenuation in resistance. The decay rate, as highlighted in the inset, progressively slowed with increasing cycle number, as evidenced by comparing the responses at the 500th, 2500th, and 4500th cycles. The sensor’s response and recovery times, measured under a 5% strain applied at 500 mm/min, are 486 ms and 492 ms, respectively (Figure 3c), demonstrating its capability for rapid sensing and restoration. Figure 3d presents the loading-unloading curves under various small strains, revealing well-separated responses for each level. Furthermore, cyclic tests at 5% strain and different stretching rates (Figure 3e) show a gradual decay in the resistance change rate over time, regardless of the applied speed. Additionally, the step response tests under four distinct strain levels in Figure 3f demonstrate not only clearly distinguishable relative resistance values but also a consistent decaying trend in resistivity across all cases. To investigate the observed degradation of the CNT sensors, we conducted 5000-cycle experiments under identical initial resistance and sensitivity conditions at strain levels of 5%, 10%, 15% and 20%, and recorded the resistance traces shown in Figure 3g. The traces indicate that as strain amplitude increases, the peak resistance decays more markedly and reaches a quasi-steady state more rapidly. For example, when the number of stretch cycles increased from 1000 to 2000, the peak resistance at 5% strain dropped from 2410 Ω to 2375 Ω, a decline of 1.45%. Under the same change in cycle count, the 10% strain condition saw the peak value fall from 2542 Ω to 2517 Ω, corresponding to a 0.98% decrease. At 15% strain, the peak decreased from 2611 Ω to 2596 Ω over the same interval, which amounts to a 0.57% reduction. By contrast, at 20% strain, the peak resistance was effectively stable after the first 1000 cycles, indicating that the sensor had reached a new equilibrium within that early cycling window. The underlying microscopic mechanisms for the observed degradation include relative sliding between CNT bundles, initiation and propagation of microcracks, and viscoelastic relaxation of the matrix; these processes lead to rapid reconfiguration of conductive pathways and the establishment of a new balance early in cycling, and larger strain amplitudes accelerate this equilibration.

**Figure 4:**
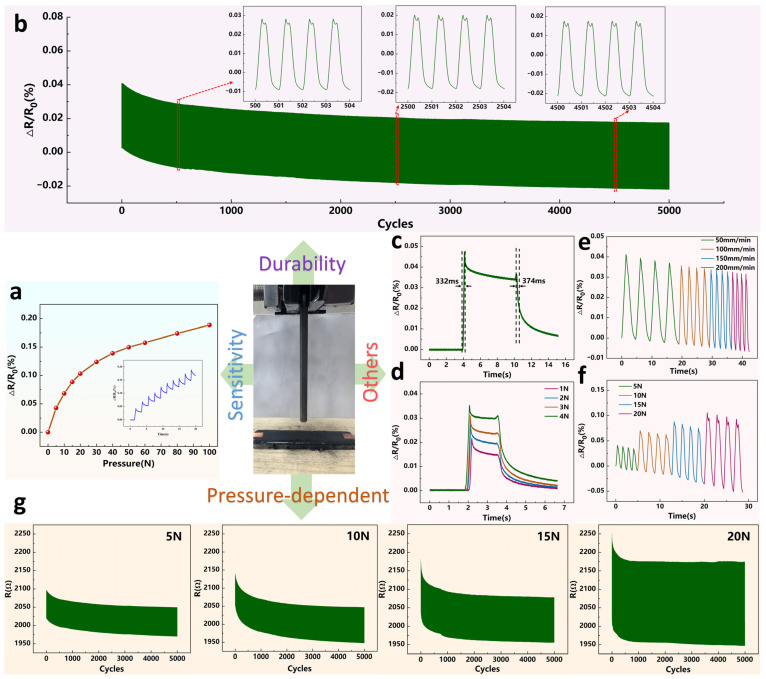
Fundamental pressure performance and data collection of the spray-coated CNT sensor. (**a**) Sensitivity curves of the sensor at 0–100 N pressure. (**b**) Durability test of the sensor. (**c**) Response time and recovery time at 5 N pressure of the sensors. (**d**) Comparison of response times under different small pressures. (**e**) Cyclic resistance response of the sensors at 5 N pressure with different pressing rates. (**f**) Step response at various pressure levels. (**g**) Collect the changes in the raw values of the cyclic tensile resistance of the sensor.

The fabricated sensors also function as pressure sensors. To ensure rigor and generality, analogous characterization was performed on spray-coated CNT devices. A flat glass platen was used to distribute force evenly and avoid stress concentration. Figure 4a presents the sensor’s sensitivity curve, demonstrating stable operation within the tested range of 0–100 N, with no abrupt signal jumps or anomalous fluctuations observed. Figure 4b shows the durability test results: after 5000 cycles under a pressure of 5 N, the device’s resistance exhibited a significant decay. The inset further compares the data from the 500th, 2500th, and 4500th cycles, indicating that the resistance decay gradually tends towards stabilization as the number of cycles increases, with the decay rate becoming progressively slower. Figure 4c illustrates the sensor’s response (332 ms) and recovery time (374 ms) when a 5 N pressure was applied at a stretching rate of 500 mm/min. This demonstrates its rapid response and resilience to external pressure stimuli. Figure 4d displays the loading/unloading curves under different pressure conditions, confirming the sensor’s excellent distinguishability for various subtle pressures. Figure 4e shows that under repeated 5 N pressure applied at different rates, the resistance consistently degrades over cycles. This indicates that varying the application rate cannot prevent fatigue-induced resistance decay. Figure 4f gives step responses under four pressure levels and shows that the relative resistance values corresponding to each pressure are clearly distinguishable while all exhibiting some degree of decay. Pressure cycling follows the same pattern as shown in Figure 4g. It should be noted that the final quasi equilibrium does not imply preserved performance: it is typically accompanied by reduced sensitivity, baseline drift and degraded repeatability.

### 3.2. LSTM–Multiscale Sequence Attention Transformer Prediction Model

Degradation sequences of sensors typically exhibit multiscale temporal components, including periodic responses, noise, and slow decay trends, rendering the degradation pattern complex. These sequences also display instability and state dependence, where prior stretch deformations influence current outputs. In this context, LSTM effectively captures local-scale phenomena such as periodic peaks, transient responses, and short-term correlations, while a sequence attention Transformer excels at modeling long-term dependencies, cross-cycle correlations, and slow trends. Combining the two enables comprehensive feature extraction from degradation sequences: LSTM first extracts window-level short-term features, which are then downsampled into tokens for the Transformer to integrate across segments and generate multi-step predictions. This design effectively mitigates recursive error accumulation and enhances prediction accuracy. By contrast, conventional self-attention assigns weights based on pointwise value similarity and thus ignores the local temporal trend of the signal, which can cause many points with similar magnitudes but different trends to be attended together and thereby interfere with capturing true degradation patterns. To address this issue, we replace self-attention with the proposed sequence attention mechanism (Figure S2), which raises the attention granularity to the subsequence level: it compares the trend of short contiguous segments composed of several consecutive time steps to identify similar fluctuations, focuses only on those subsequences that exhibit the same periodic features within the local window, and then processes features from these matched subsequences. This approach accurately extracts locally time-varying signals while retaining the global information of the overall degradation curve, making it particularly suitable for the periodic fluctuation characteristics of flexible sensor degradation (Figure S3). From a data perspective, the hierarchical LSTM plus sequence attention Transformer better handles the multiscale structure, instability and state dependence of degradation data. The framework hyperparameters are listed in Table S1.

To address the sample scarcity that commonly arises from limited device usage time, we apply a generative adversarial network to augment the time series of degradation sequences. The GAN learns the temporal distribution of the original sensor data and generates high-quality pseudo samples, thereby expanding the training set and introducing corresponding fluctuation patterns so that the augmented samples share the same degradation trends as the real data. This practice helps the model more comprehensively capture periodic variations and subtle degradation trends and effectively reduces overfitting risk caused by insufficient samples, thereby improving the robustness and generalization capability of the prediction and compensation model in low-sample scenarios. Because this study focuses on the degradation trend of flexible sensors and because the peak values of resistance are both closely related to various practical operating conditions and more directly reflect the degradation process, we introduce a peak detection mechanism to extract information at these critical instants. By locating the peak in each cycle, we emphasize the sensor’s performance change under high strain or high load and achieve automatic alignment of similar phases in the time series, which strengthens the model’s ability to discriminate degradation patterns. As salient features, peaks also suppress background noise and improve the representativeness and separability of the input data, thereby helping to increase the accuracy and robustness of the subsequent prediction model. Based on the complexity of degradation sequences and the scarcity of samples, we propose a prediction framework that integrates GAN-based data augmentation and peak detection with an LSTM–sequence attention Transformer.

**Figure 5:**
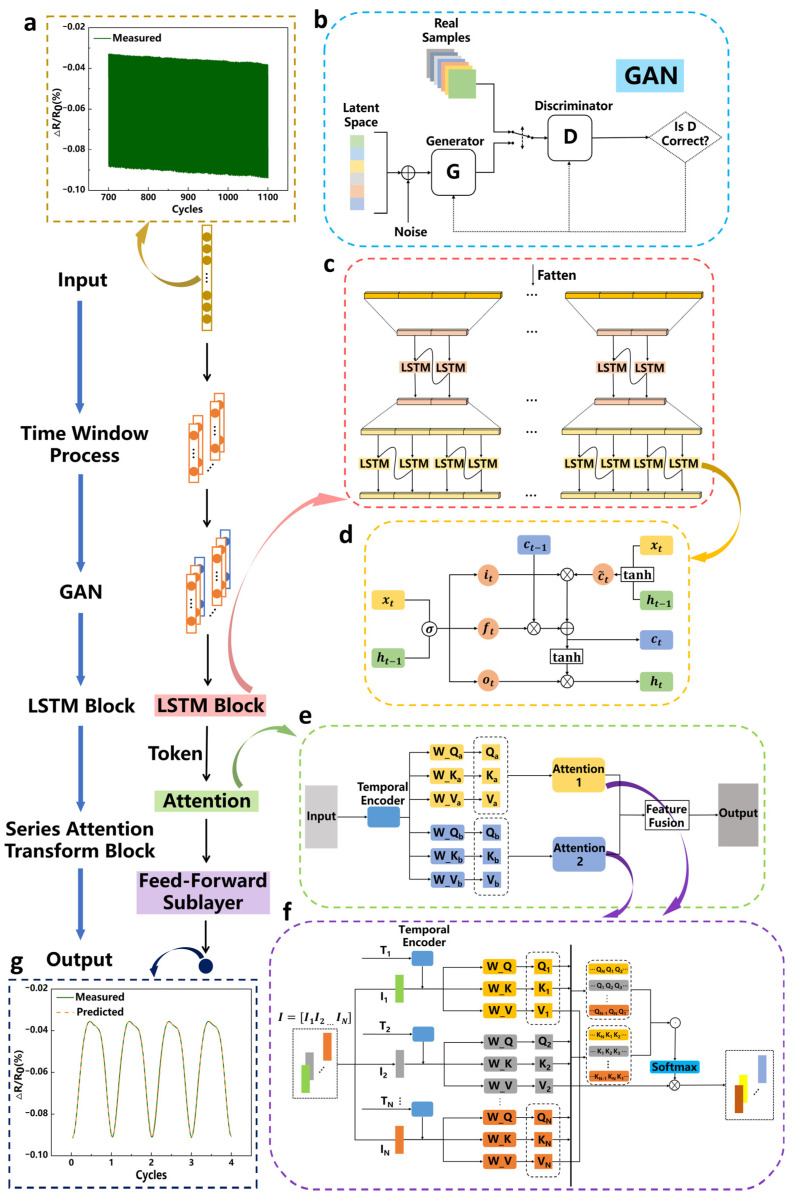
Overall architecture of the model. (**a**) System input data of the model. (**b**) GAN workflow. (**c**) LSTM layer architecture. (**d**) LSTM cell module. (**e**) Schematic of the multiscale attention mechanism. (**f**) Schematic of the sequence attention mechanism. (**g**) System prediction output of the model.

Figure 5 illustrates the overall architecture of the prediction model. For the degradation prediction task of flexible sensors, the model takes the preprocessed resistance time series as input (Figure 5a). First, a sliding time window segments the long series into fixed-length subsequences so that each subsequence serves as the basic processing unit for downstream modules. Next, a GAN is applied for data augmentation (Figure 5b) to mitigate overfitting in small-sample regimes and to improve model generalization. The augmented subsequences are then fed into the LSTM module (Figure 5c) and processed by the LSTM cell (Figure 5d). The LSTM recursively models short-term dependencies within each segment, and the resulting per-segment hidden-state sequences are aggregated at the segment level to produce a fixed-length vector. These vectors are linearly projected to the Transformer embedding dimension, which performs tokenization, and positional encodings are added to each token to preserve intersegment order. The token sequence is then input to the multiscale sequence attention Transformer composite module (Figure 5e,f). Sequence-level attention measures similarity at the subsequence scale and aligns tokens with similar local trends, while the Transformer encoder captures long-range dependencies in the degradation trend. Finally, the fused features are mapped by the output layer to future degradation indicators, yielding accurate predictions of flexible sensor performance as shown in Figure 5g.

### 3.3. Baseline Comparison and Ablation Study

The proposed method aims to predict the performance degradation of flexible sensors and then compensate for measurement error based on the predicted degradation trajectory. Therefore, the model must predict subsequent degradation as accurately as possible from the training data. Root mean square error, mean absolute error and the coefficient of determination are used to evaluate the model’s predictive capability. Root mean square error (RMSE)and mean absolute error (MAE) are two common measures of the discrepancy between actual and predicted values and are employed here to compare the performance of different prediction models. Smaller *MAE* and *RMSE* indicate more accurate predictions and lower error. The coefficient of determination *R* squared gauges the explanatory power of the predictor, with larger *R*-squared indicating better prediction and an *R*-squared equal to one indicating a perfect match to the ground truth. The evaluation metrics are defined as follows.

(2) $$RMSE=\sqrt{\frac{1}{m}\sum_{i=1}^{m}{\left(y_{i}-\hat{y_{i}}\right)}^{2}}$$

(3) $$MAE=\frac{1}{m}\sum_{i=1}^{m}\left|y_{i}-\hat{y_{i}}\right|$$

(4) $$\bar{y}=\frac{1}{m}\sum_{i=1}^{m}\hat{y_{i}\;}$$

(5) $$R^{2}=1-\frac{\sum_{i=1}^{m}{\left(y_{i}-\hat{y_{i}}\right)}^{2}}{\sum_{i=1}^{m}{\left(y_{i}-\bar{y}\right)}^{2}}$$

Here $\hat{y_{i}}$ denotes the actual value, $y_{i}$ denotes the predicted value, and m denotes the number of ground truth measurements. To compare predictive approaches and meet practical needs, this work contrasts multi-step prediction with long-term prediction, as shown in Figure S4, and ultimately adopts multi-step prediction. By incorporating the latest observations into each prediction window through a correction and feedback mechanism, multi-step prediction effectively suppresses error accumulation that grows with the prediction horizon. Given that sensor degradation typically evolves gradually, multi-step forecasts over short horizons provide continuous degradation curves that better match maintenance and early warning requirements. Restricting sequence length during training and inference also reduces training instability and computational cost. Taken together, multi-step prediction achieves a favorable balance among predictive accuracy, interpretability and efficiency, aligning with the engineering objectives of this study.

**Figure 6:**
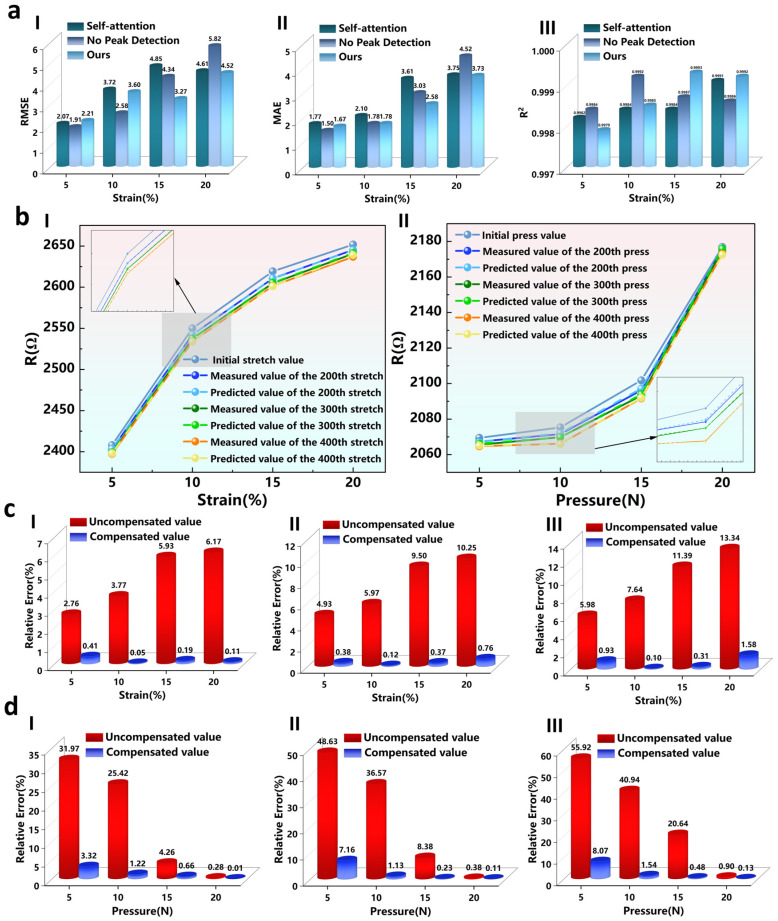
(**a**) Comparison of three model evaluation metrics under different strain levels, (I) MAE, (II) RMSE, (III) R^2^. (**b**) Comparison of measured peaks and predicted peaks at different cycle counts, (I) Strain conditions, (II) Pressure conditions. (**c**) Comparison of relative errors before and after compensation under strain conditions, (I) 200 cycles, (II) 300 cycles, (III) 400 cycles. (**d**) Comparison of relative errors before and after compensation under pressure conditions, (I) 200 cycles, (II) 300 cycles, (III) 400 cycles.

To rigorously justify the structural complexity of the proposed framework, it is essential to evaluate its performance against standard baseline methods. During the preliminary stages of this study, various simpler models, including classical machine learning approaches (e.g., SVR), were evaluated. However, these simpler methods proved inadequate for capturing the highly non-linear, history-dependent degradation and severe hysteresis of the CNT sensors, resulting in unacceptably high predictive errors. Therefore, we utilized the standard Transformer architecture with conventional pointwise self-attention as our primary deep learning baseline. Furthermore, to validate the contribution of each module in the proposed prediction framework, we designed ablation experiments that compare predictions on held-out degraded sequences to quantify component-wise contributions. All experiments use the best hyperparameters selected on the validation set. Concretely, we removed the GAN data augmentation module, the peak detection mechanism and the sequence attention mechanism in turn to obtain three comparison models: a model without GAN to evaluate the effect of augmentation under small sample conditions; a Transformer model that uses conventional self-attention instead of sequence attention to test the advantage of sequence level feature extraction; and a model without peak detection to analyze the impact of extrema information on degradation forecasting. Ablation analyses are performed on tensile cyclic data by training each model on extracted degraded subsequences at different strain levels and reporting RMSE, MAE and R-squared to judge prediction performance. The systematic evaluation of each component’s contribution to predicting resistance decay in flexible sensors is summarized in Figure 6a and Table 1.

Figure 6a and Table 1 show that as strain increases from 5% to 20%, the overall data volume grows and the model’s generalization correspondingly improves. The proposed model, which incorporates GAN data augmentation, peak detection and a sequence attention Transformer, outperforms the Transformer using only self-attention on all metrics except the RMSE at 5% strain. Relative to the baseline without GAN, RMSE and MAE decrease substantially at every strain level, which demonstrates that data augmentation effectively alleviates the small-sample problem. Given that these evaluation metrics are computed exclusively on strictly sequestered, real measurement data, the substantial reduction in error provides robust empirical proof that the GAN successfully learned the underlying physical degradation trends without introducing harmful artificial bias. However, at the smallest strain level (5%), the proposed model yields slightly higher error than the peak-detection–removed variant, with RMSE = 2.21 and MAE = 1.67 compared with RMSE = 1.91 and MAE = 1.50 for the model without peak detection; a similar pattern appears at 10% strain where the proposed model’s RMSE = 3.60 is worse than the no-peak model’s RMSE = 2.58. This behavior can be explained by the extremely small-sample regime, in which the peak detection mechanism is more sensitive to noise and can overemphasize extrema at the expense of learning other sequence patterns. As strain and therefore sample size increase, the synergistic effect of peak detection and sequence attention emerges and our method surpasses all comparison models at medium and high strain levels. Figures S5–S8 compare the resistance predictions of the four models under different strain levels using only the first four cycles from each sample. The plots show that at 5% strain, the proposed model’s prediction closely follows the measured trace overall but is slightly inferior to the variant without peak detection at a few extrema. At 10%, 15% and 20% strain, the proposed model outperforms both the no-peak variant and the Transformer that uses standard self-attention in terms of amplitude and phase alignment, yielding a higher degree of overlap with the measured curves, especially at peak and trough positions and in overall waveform shape. By contrast, the model without GAN exhibits the largest errors and the most evident waveform deviations across all strain levels, which demonstrates the clear benefit of data augmentation in small-sample regimes. In summary, the ablation results confirm that sequence attention and peak detection jointly improve degradation curve fitting when the sample size is sufficient, while in extremely low-sample scenarios such as 5% strain, the local sensitivity of peak detection can lead to overemphasis of certain extrema.

### 3.4. Establishment and Validation of the Predict and Compensate Framework

To compensate for measurement error based on predicted degradation and to validate the compensation effect, we compiled peak resistance values of the sensor under different strain levels and different pressures (Figure S9), including the initial peaks, the measured peaks after 200, 300 and 400 cycles, and the corresponding peaks predicted by the proposed model as shown in Figure 6b. The results indicate that stretch deformation cycles cause peak attenuation and that the degree of degradation increases with cycle count. Peak values also rise with increasing strain but the growth rate decreases as strain increases, producing a curve that tends toward flattening. Pressure cycling similarly induces degradation, with peak values increasing with applied pressure. Unlike the strain case, the growth rate with respect to pressure rises as pressure increases, resulting in greater amplitude growth over the 5–20 N range. The different trends arise because strain is a dimensionless relative deformation while pressure is an absolute force, and the two cannot be converted linearly. Moreover, stretch and applied pressure act differently during fatigue, producing distinct electrical sensitivity and degradation rates. Because sensor measurements in application are determined from the strain or pressure to resistance relationship, we implement compensation by replacing the initial peak curve with the model-predicted peak curve corresponding to a specific cycle count. In other words, the predicted peak curve for the given cycle is used as the updated reference so that the degraded measured resistance and the substituted reference agree closely. This output level baseline update corrects for errors introduced by peak attenuation and effectively restores the sensor measurement accuracy. By fitting analytic functions to the initial peak curve and to the model-predicted peak curve, two corresponding closed-form expressions are obtained that permit estimation of the peak degradation at any strain or pressure within the sensor’s operational range. These two functions allow computation of the discrepancy between measured values and the peak prediction before and after compensation, thereby quantifying the practical relative error. For example, at 400 stretch cycles and an actual strain of 15%, the measured resistance is 2601.4 Ω. Interpreting this resistance against the initial uncycled peak curve yields an inferred strain of 13.29% and a relative error of (15% − 13.29%)/15% ≈ 11.39%. Interpreting the same resistance against the 400-cycle predicted peak curve yields an inferred strain of 15.04% and reduces the relative error to about 0.31%. This example demonstrates that fitting and then substituting the predicted peak curve for the corresponding cycle substantially reduces the interpretation error caused by peak attenuation.

To validate the compensation effect, we computed and summarized pre- and post-compensation relative errors for cycles 200, 300 and 400 at strain levels of 5%, 10%, 15% and 20% and at pressure levels of 5 N, 10 N, 15 N and 20 N. Figure 6c shows that pre-compensation relative error increases as strain grows from 5% to 20% and that larger cycle counts further increase error, reflecting stronger output drift and measurement bias at higher strain and with more cycles. Figure 6c reveals a different error versus load pattern under pressure because the resistance versus load response in Figure 6d has a distinct shape. The pressure response is relatively flat in the low-load region, so an identical absolute resistance decrease corresponds to a larger relative error there, which amplifies error at small pressures and implies the sensor is better suited to operate under small strain or larger pressure. In all cases, increasing the cycle count still aggravates error. In contrast, after applying the proposed compensation procedure, relative errors drop markedly across operating conditions. Under strain, the relative error ranges from 2.76% to 13.34% is reduced to about the 1% level, representing a typical reduction to roughly 85–90% of the uncompensated values. Under pressure, the uncompensated errors are larger; at 5 N the pre-compensation relative errors after 200, 300 and 400 cycles reach 31.97%, 48.63% and 55.92% respectively, and after compensation, these points fall to 3.32%, 7.16% and 8.07% in the same order. Although the compensated absolute errors under pressure remain slightly higher than in the strain cases, the relative reduction ratio is still about 85%. These results indicate that the compensation mechanism effectively corrects the nonlinear errors that grow with strain or load and thereby substantially improves measurement reliability and accuracy for flexible sensors in practical applications. To ensure statistical reliability, it should be noted that all reported compensation results are the statistical mean of 5 independent runs with different random initializations. The variance across these runs is extremely narrow (e.g., the standard deviation of relative errors remains below ± 3%), which rigorously validates the stability of the compensation mechanism.

### 3.5. Applications: Gesture and Region Recognition

**Figure 7:**
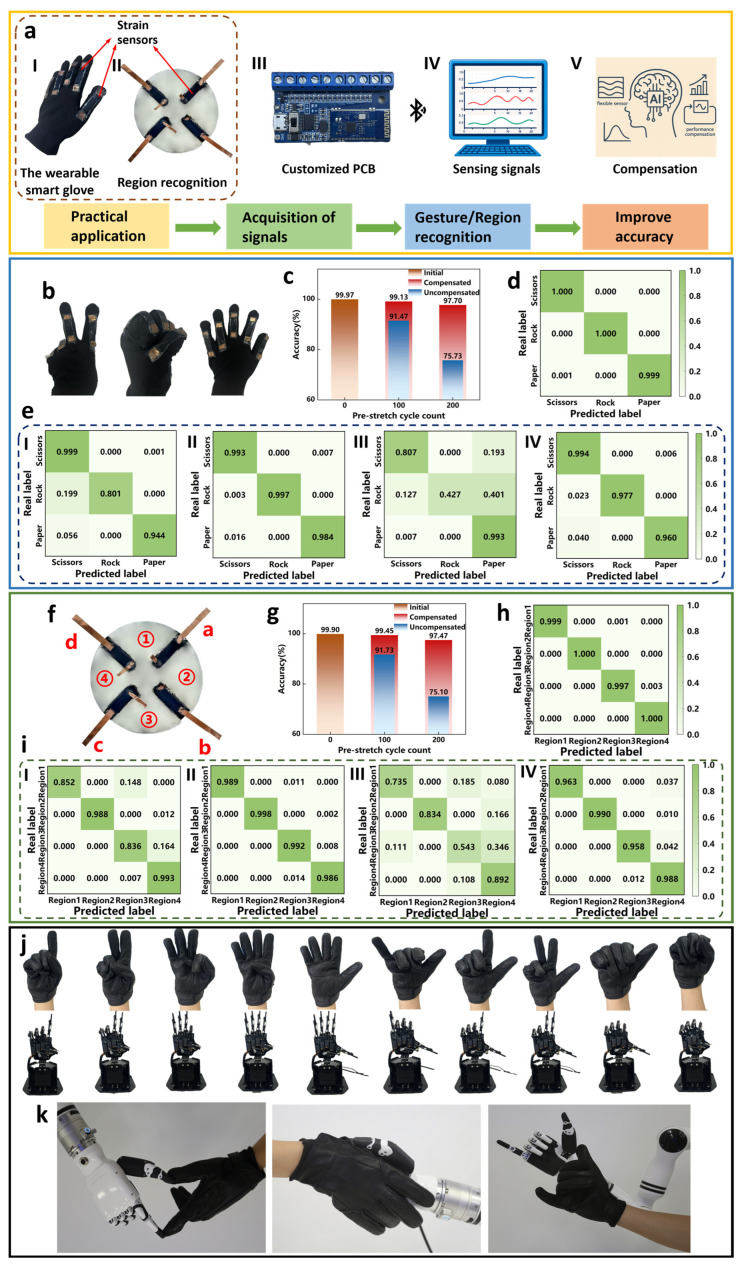
Compensation mechanism applied to improve recognition accuracy in gesture recognition and region recognition. (**a**) System workflow schematic, (I) Smart glove performs gestures, (II) Schematic of region recognition application, (III) Acquisition board records voltages from flexible sensors in the application, (IV) MLP-based gesture and region recognition from the acquired voltage signals, (V) Use of the compensation mechanism to enhance recognition accuracy. (**b**) The three target gestures to be recognized. (**c**) Recognition accuracy comparison after different numbers of grips: initial, after compensation, and without compensation. (**d**) Initial confusion matrix of the gesture recognition system. (**e**) Confusion matrices for gesture recognition after different numbers of grips: (I) After 100 grips, (II) after 100 grips following compensation, (III) after 200 grips, and (IV) after 200 grips following compensation. (**f**) Sensor and region numbering in the region recognition application, where a–d represent the four conductive interfaces, and 1–4 represent the four regions. (**g**) Recognition accuracy comparison after different prestretch counts: initial, after compensation, and without compensation. (**h**) Initial confusion matrix of the region recognition system. (**i**) Confusion matrices for region recognition after different prestretch counts, (I) After 100 stretches, (II) After 100 stretches following compensation, (III) After 200 stretches, (IV) After 200 stretches following compensation. (**j**) Smart glove controlling a robotic gripper to perform various gestures. (**k**) Smart glove controlling a robotic arm.

To further evaluate the effectiveness and generality of the proposed predict and compensate framework, we developed a smart glove gesture recognition system and a region recognition system that integrate the flexible deformation sensors described above, as shown in Figure 7a. Five sensors are mounted on the finger joints of the glove to form an integrated sensor array. During finger bending, each sensor undergoes deformation and different gestures produce different strain levels and corresponding relative resistance changes. The sensor array transmits the acquired voltage signals for different gestures to a computer via Bluetooth (Figure S10), and a convolutional neural network is trained on these voltage patterns to perform gesture recognition.

Because sensor outputs drift and sensitivity degrades with use, the input distribution to the downstream classifier departs from the training distribution, which degrades the classifier decision boundaries and reduces recognition accuracy. Applying the proposed predict and compensate framework enables real-time multi-step prediction of sensor outputs and maps degraded signals back to decision values close to the training distribution, thereby correcting bias and amplitude attenuation caused by degradation and substantially restoring recognition accuracy and system robustness under long-term operation. To validate the compensation effect in the gesture recognition system, we ran comparative experiments at different fatigue levels (initial, 100 grips and 200 grips), ensuring that each sensor was rested sufficiently before each grip cycle to simulate realistic fatigue scenarios. The three target gestures are presented in Figure 7b. As shown in Figure 7c, after 100 cycles, the uncorrected recognition accuracy drops from 99.97% to 91.47% and is recovered to 99.13% ± 0.27% after compensation; after 200 cycles, the uncorrected accuracy falls to 75.73% and is improved to 97.70% ± 0.42% with compensation. The corresponding normalized confusion matrices in Figure 7d indicate that the predict and compensate mechanism substantially increases diagonal recall rates and reduces interclass confusion. In the region recognition system, four flexible sensors are arranged on an Ecoflex substrate and the sensing area is partitioned into four subregions labeled 1 through 4 (Figure 7e). Each region is stimulated by applying a 5% outward strain along the midline, eliciting pronounced resistance changes in the sensors on either side. Consequently, stimulating different regions produces distinct resistance patterns across the four sensors. The recorded four-channel voltage signals (Figure S11) are input to an MLP, which, after training, identifies the specific region where strain occurred and thereby accomplishes region recognition. Under the same comparative conditions used for the gesture experiments, Figure 7f shows that after 100 and 200 stretch cycles, the uncorrected recognition accuracies fall to 91.73% and 75.10%, respectively, whereas introducing the predict and compensate framework restores accuracies to 99.45% ± 0.29% and 97.47% ± 0.35%, markedly outperforming the uncorrected results. The confusion matrix in Figure 7g likewise indicates accurate identification of all regions with substantially reduced misclassification.

Overall, the proposed predict-and-compensate framework leverages predicted sensor degradation trends to dynamically adjust gesture-specific decision thresholds based on fatigue level. This maintains alignment between the input distribution and classifier decision boundaries, significantly enhancing recognition accuracy and robustness during prolonged cyclic use. However, the algorithm offers limited recovery for extreme faults, causing irreversible structural damage. In this study, the developed smart glove not only acquires finger bending information in real time and achieves accurate gesture recognition after prediction and compensation, but also supports remote control of a robotic gripper (Figure 7h) and a robotic arm (Figure 7i) and provides game control functionality (Figure S12). Users can remotely operate the gripper and arm opening/closing and rotation through a set of gestures to realize human–machine interaction, a capability with broad application potential in intelligent manufacturing, rehabilitation medicine and robotic interaction.

## 4. Discussion

### 4.1. Operational Strain Regime and Limitations

While the proposed deep-learning-based predict-and-compensate framework demonstrates exceptional performance in restoring degraded sensor signals, it is necessary to clarify its intended operational regime. The current methodology focuses primarily on the low-to-medium strain range, which effectively encompasses the typical deformations encountered in our target applications, such as spatiotemporal human–machine interaction and regional strain mapping. Within this specific regime, sensor degradation is predominantly governed by gradual microcrack initiation and the viscoelastic relaxation of the polymer matrix, resulting in continuous and mathematically trackable fatigue patterns.

However, in practical scenarios where flexible sensors are subjected to significantly larger deformations (e.g., 50% or up to 100% strain), the underlying physical degradation mechanisms shift fundamentally. Extreme stretching induces severe and irreversible structural destruction, including complete conductive network rupture, massive crack propagation, and interfacial delamination. These phenomena typically lead to highly nonlinear, abrupt, and catastrophic signal losses. Such abrupt structural failures deviate significantly from the gradual fatigue trajectories modeled in this study, and they may exceed the extrapolative capabilities of the current data-driven framework. Therefore, generalizing this method to large-strain and extreme-deformation applications remains a limitation. Future research should consider constructing broader, multi-regime degradation datasets and developing adaptive switching algorithms to accommodate these severe structural failures.

Conversely, at the lower boundary of the operational regime (e.g., extreme low-sample or 5% minimal strain conditions), the system faces a different limitation. In these scenarios, the absolute degradation amplitude is minimal, and the signal-to-noise ratio (SNR) is inherently low. As observed in our ablation studies, aggressive feature extraction modules—specifically the peak detection mechanism—can exhibit heightened sensitivity to background noise in such low-SNR environments, occasionally overemphasizing noise spikes and leading to minor predictive fluctuations. Although the absolute prediction error remains exceptionally small (e.g., RMSE = 2.21) and does not compromise practical operational stability, this highlights a boundary limitation of the current architecture. Developing adaptive algorithms that dynamically reduce the weighting of extrema-based features in extremely low-SNR regimes remains an important objective for future refinement.

### 4.2. Perspectives on Physical Interpretability

Although the proposed compensation framework relies on a data-driven architecture to fit sensor degradation behavior, its structural design is fundamentally inspired by the underlying physical mechanisms of CNT-based sensors. Specifically, the hybrid nature of the network maps effectively to the dual-timescale characteristics of sensor degradation. The LSTM module, with its recurrent memory mechanism, is intrinsically suited for capturing short-term, history-dependent dynamic features, which are physically analogous to the instantaneous viscoelastic relaxation and delayed recovery of the elastomeric substrate. Conversely, the Transformer module is employed to capture global, long-range dependencies, mirroring the continuous, long-term baseline drift caused by cumulative microcrack propagation and macroscopic conductive network reconfiguration.

We acknowledge that integrating physics-informed neural networks (PINNs) represents the ultimate paradigm for robust sensor modeling. However, defining precise and universal partial differential equations (PDEs) to continuously describe the dynamic, chaotic reconstruction of nanoscale CNT networks under complex ambient conditions remains an unresolved challenge in solid mechanics. Consequently, formulating a strict, purely physics-driven constraint is currently difficult without introducing overly simplified assumptions. Therefore, our data-driven approach, guided by physics-inspired architectural choices, currently offers a highly practical and deployable solution. In future iterations, we plan to explore the integration of soft physical constraints, such as incorporating phenomenological fatigue damage models or hyperelastic strain energy functions as regularization terms within the loss function, to further enhance the physical interpretability and generalizability of the learning framework.

### 4.3. Computational Efficiency and Real-Time Feasibility

For any “online” compensation framework, computational efficiency is a prerequisite to ensure real-time sensing and accurate downstream recognition. Although the proposed algorithmic architecture integrates GAN, LSTM, and Transformer modules, it is structurally optimized to minimize inference latency within a PC-based software environment (Python 3.7.6 and PyTorch 1.13.0). It is important to note that the GAN module is employed strictly for offline time-series augmentation during the training phase. Consequently, it is completely decoupled from the deployment stage and does not contribute to any computational overhead during online monitoring.

The online inference pipeline consists solely of the pre-trained LSTM and the Sequence-Level Attention Transformer. Traditional Transformer architectures often suffer from quadratic computational complexity with respect to the sequence length. However, our sequence-level attention mechanism aggregates pointwise historical data into segment-level tokens before computing self-attention. This design dramatically reduces the effective sequence length, thereby significantly decreasing both the computational footprint and memory usage.

To quantitatively evaluate the real-time performance, we measured the inference latency of the compensation pipeline. On a standard host workstation equipped with an Intel Core i9-10900 (2.8 GHz) processor and 25.9 GB of memory, a single forward pass—from receiving the degraded signal sequence to calculating the compensated output—requires approximately 5 ms. In our wearable applications, the data acquisition board operates at a sampling frequency of 500 Hz, meaning new data points arrive every 2 ms. More importantly, typical human gesture dynamics occur on a physiological timescale of 100 to 300 ms. Because the algorithm’s inference latency is in the single-digit millisecond range, it processes data orders of magnitude faster than the timescale of human motion. Therefore, the proposed framework performs synchronized signal restoration without introducing any perceptible lag, fully satisfying the real-time requirements for online human–machine interaction systems.

### 4.4. Long-Term Stability in Practical Applications

To fully address the long-term operational stability of the proposed framework, it is essential to contextualize the gesture recognition results with the fundamental sensor durability. As previously demonstrated, the bare CNT sensors underwent continuous cyclic testing for up to 5000 cycles, establishing a robust physical baseline for the predictive model. In the practical smart glove demonstration, although the recognition evaluation highlighted 100 and 200 continuous, large-angle grip cycles, these motions induce substantial localized strain and microstructural network reconfiguration, pushing the system into an advanced fatigue state. The severity of this degradation is reflected in the uncompensated accuracy dropping steeply to 75.73%.

The framework’s ability to restore this accuracy to 97.70% under such severe attenuation confirms its efficacy. Moreover, the integration of GAN-based data augmentation during the offline training phase exposed the model to simulated extreme-drift trajectories equivalent to thousands of operation cycles. This generative prior knowledge ensures that the LSTM-Transformer architecture is not strictly bounded by the short-term calibration limits, but possesses the robust extrapolative capacity required to suppress long-term baseline drift in extended wearable applications.

### 4.5. Model Generalization and Cross-Sensor Applicability

A critical requirement for any data-driven compensation strategy is its generalization capability across different sensors and users. It is important to emphasize that the proposed predict-and-compensate framework targets the intrinsic physical degradation of the CNT sensors (e.g., structural fatigue and baseline drift), which is a device-level characteristic independent of the user. By effectively normalizing the hardware drift, the framework inherently mitigates cross-user variations in downstream tasks. Furthermore, the cross-sensor generalization of the model was validated through its deployment in the multi-sensor arrays of the smart glove (five sensors) and the region recognition platform (four sensors). Despite the inevitable variations in fabrication tolerances, initial resistances, and localized fatigue rates among these individual sensors, the proposed framework consistently mapped their degraded outputs back to the original baseline distributions. The successful recovery of recognition accuracy in these complex multi-sensor systems provides robust evidence that the algorithm generalizes well across different individual sensors without requiring sensor-specific retuning.

## 5. Conclusions

This study investigates the performance degradation of flexible CNT strain sensors induced by long-term use and repeated stretching, and proposes a systematic approach integrating experimental data acquisition, generative augmentation, and deep learning-based predictive compensation. First, degradation data under multiple strain and pressure conditions were collected experimentally, revealing resistance drift, hysteresis, and quasi-steady-state characteristics during fatigue, which provide a foundation for subsequent modeling. Second, a hybrid framework was developed, centered on GAN-based data augmentation, LSTM, and a multi-scale sequential attention Transformer, capable of capturing multi-scale temporal dependencies and subsequence trend similarities with limited samples, thereby enabling accurate prediction of sensor degradation and output compensation. Experiments demonstrate that the framework outperforms baseline models across various strain levels, reducing compensated prediction errors to approximately 1% and significantly mitigating originally large errors. In application validation, the compensation mechanism was integrated into MLP-based gesture recognition and region recognition systems, achieving high-precision identification of three gestures and multiple regions, confirming the framework’s practical effectiveness and generalization capability. After prolonged cyclic fatigue, compensation markedly restored recognition performance: for instance, following 200 fatigue cycles, gesture recognition accuracy recovered from 75.73% to 97.70%, and region recognition accuracy from 75.10% to 97.47%, substantially enhancing system robustness and stability. In summary, this study deepens the understanding of degradation mechanisms in flexible CNT sensors and validates a practical predictive compensation strategy to improve their long-term stability in wearable health monitoring and human–computer interaction.

## Figures and Tables

**Table 1:** Summary of evaluation metrics for the four models under different strain levels.

Strain	5%			10%			15%			20%		
	RMSE	MAE	R^2^	RMSE	MAE	R^2^	RMSE	MAE	R^2^	RMSE	MAE	R^2^
No GAN	31.11	26.34	0.5915	65.95	55.34	0.5123	86.06	69.58	0.5113	109.36	92.82	0.5141
Self-attention	2.07	1.77	0.9982	3.72	2.10	0.9984	4.85	3.61	0.9984	4.61	3.75	0.9991
No Peak Detection	1.91	1.50	0.9984	2.58	1.78	0.9992	4.34	3.03	0.9987	5.82	4.52	0.9986
Ours	2.21	1.67	0.9979	3.60	1.78	0.9985	3.27	2.58	0.9993	4.52	3.73	0.9992

## Data Availability

The original contributions presented in the study are included in the article/Supplementary Material; further inquiries can be directed to the corresponding author.

## Supplementary Materials

The following supporting information can be downloaded at https://www.mdpi.com/article/10.3390/mi17040496/s1: Detailed data on the additional cyclic stretching tests, comparative analyses of attention mechanisms and prediction types, model performance under varying strains and pressures, sensor signal variations for gesture and region recognition, application demonstrations in game control, dataset sampling methodology, and framework hyperparameters are comprehensively presented in the Supporting Information (Figures S1–S13, Table S1).
