# Supplementary material for: Deep Learning-Based Prediction and Compensation of Performance Degradation in Flexible Sensors
Source: Micromachines (Basel). 2026 Apr 18;17(4):496. doi: 10.3390/mi17040496 (PMC13118224; doi:10.3390/mi17040496)
Supplement: Supplementary file 1 [file micromachines-17-00496-s001.zip › micromachines-4277683-supplementary.pdf]

Supplementary Materials for

# Deep Learning-Based Prediction and Compensation of Performance Degradation in Flexible Sensors

**Zhiyuan Wang, Tong Zhang *, Luyang Zhang, Xiao Wang, Youli Yao, Qiang Liu, Yijian Liu and Da Chen ***

College of Electronic and Information Engineering, Shandong University of Science and Technology,

Qingdao 266590, China

* Correspondence: tonging@sdust.edu.cn (T.Z.); chenda@sdust.edu.cn (D.C.)

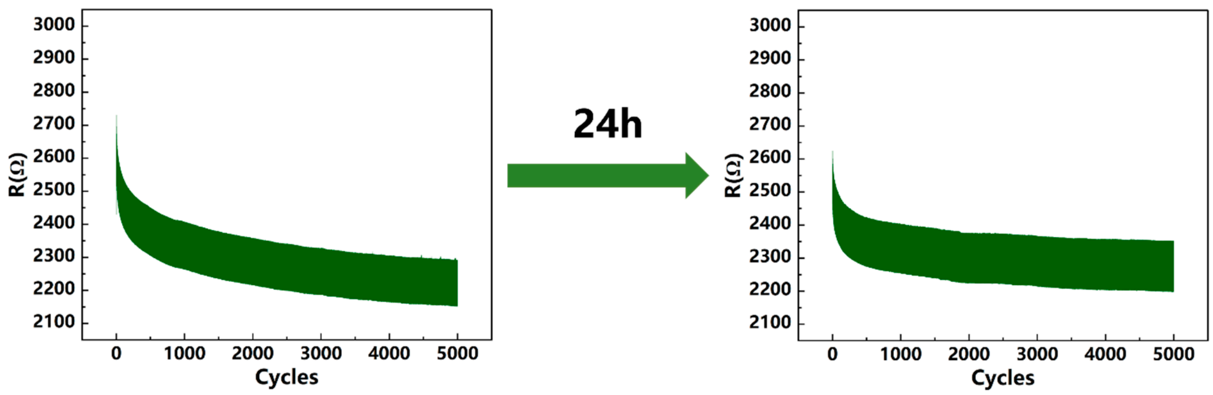


Figure S1. After 24 hours the sensor underwent an additional 5000 stretching cycles and the device still exhibited a performance degradation trend.

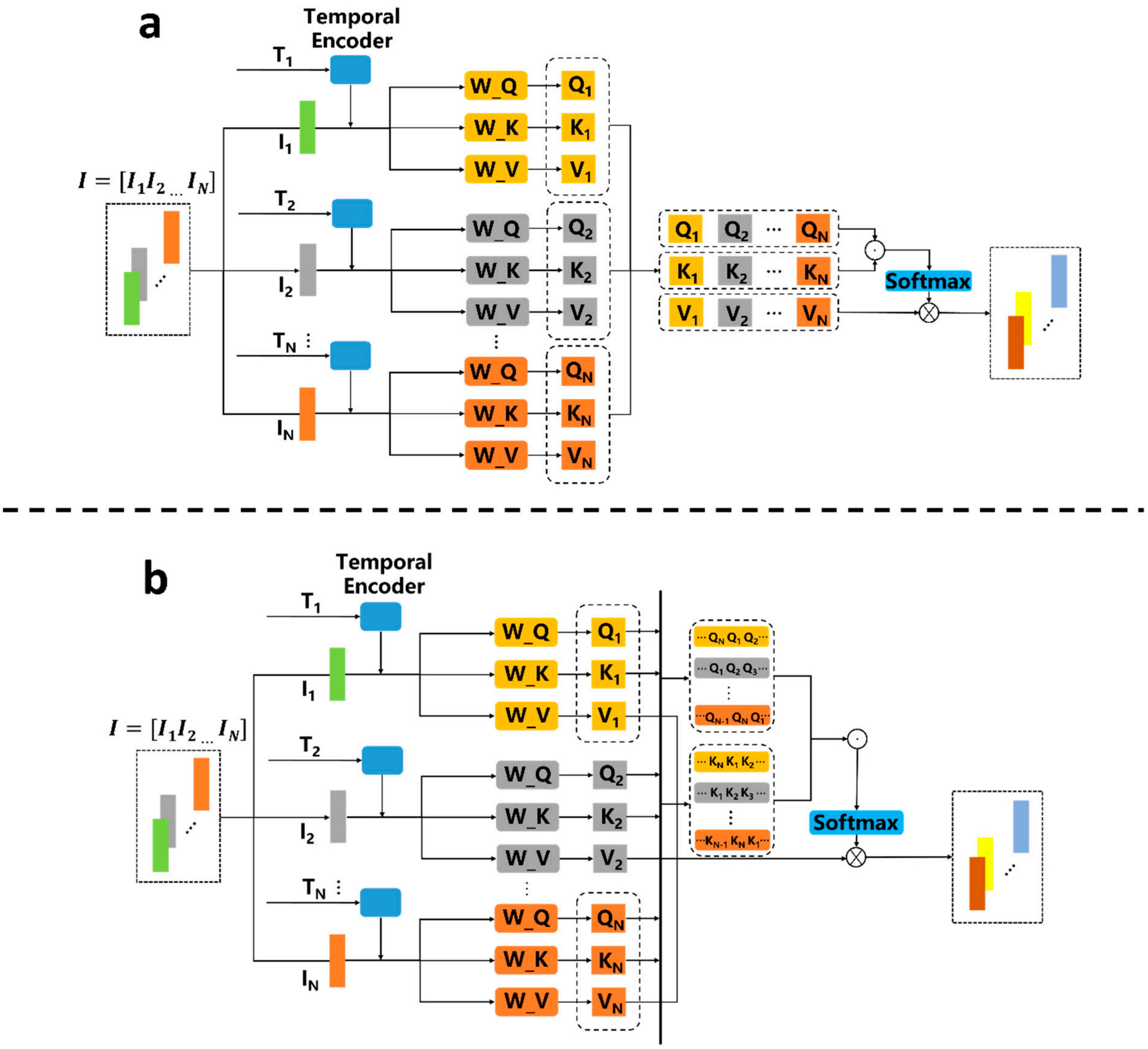


Figure S2. Difference between conventional self-attention and sequence attention. (a) conventional self-attention, (b) sequence attention.

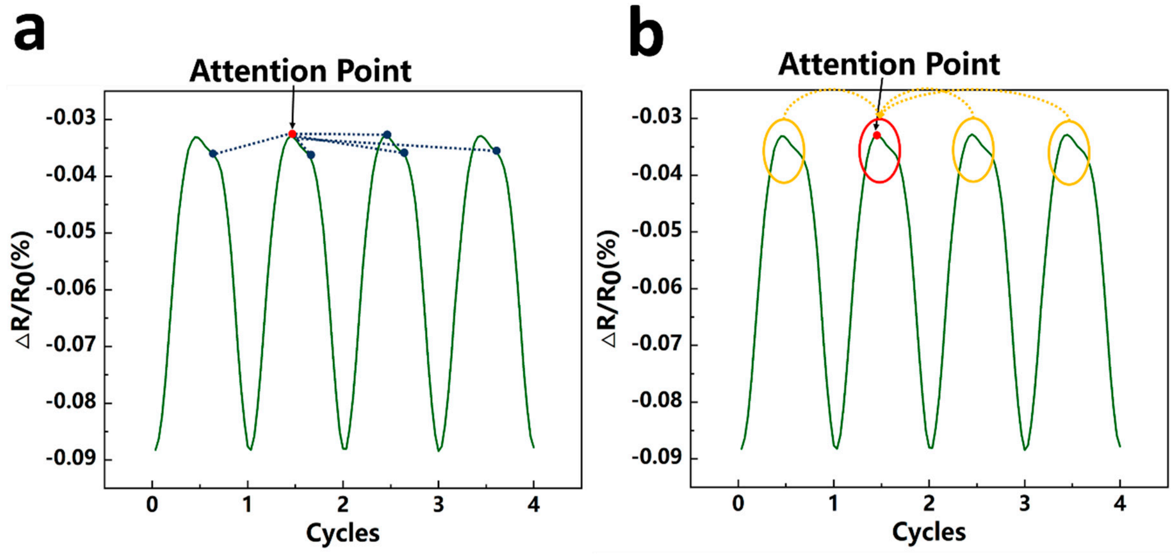


Figure S3. Comparison of two prediction mechanisms. (a) Multi step prediction, (b) Long term prediction.

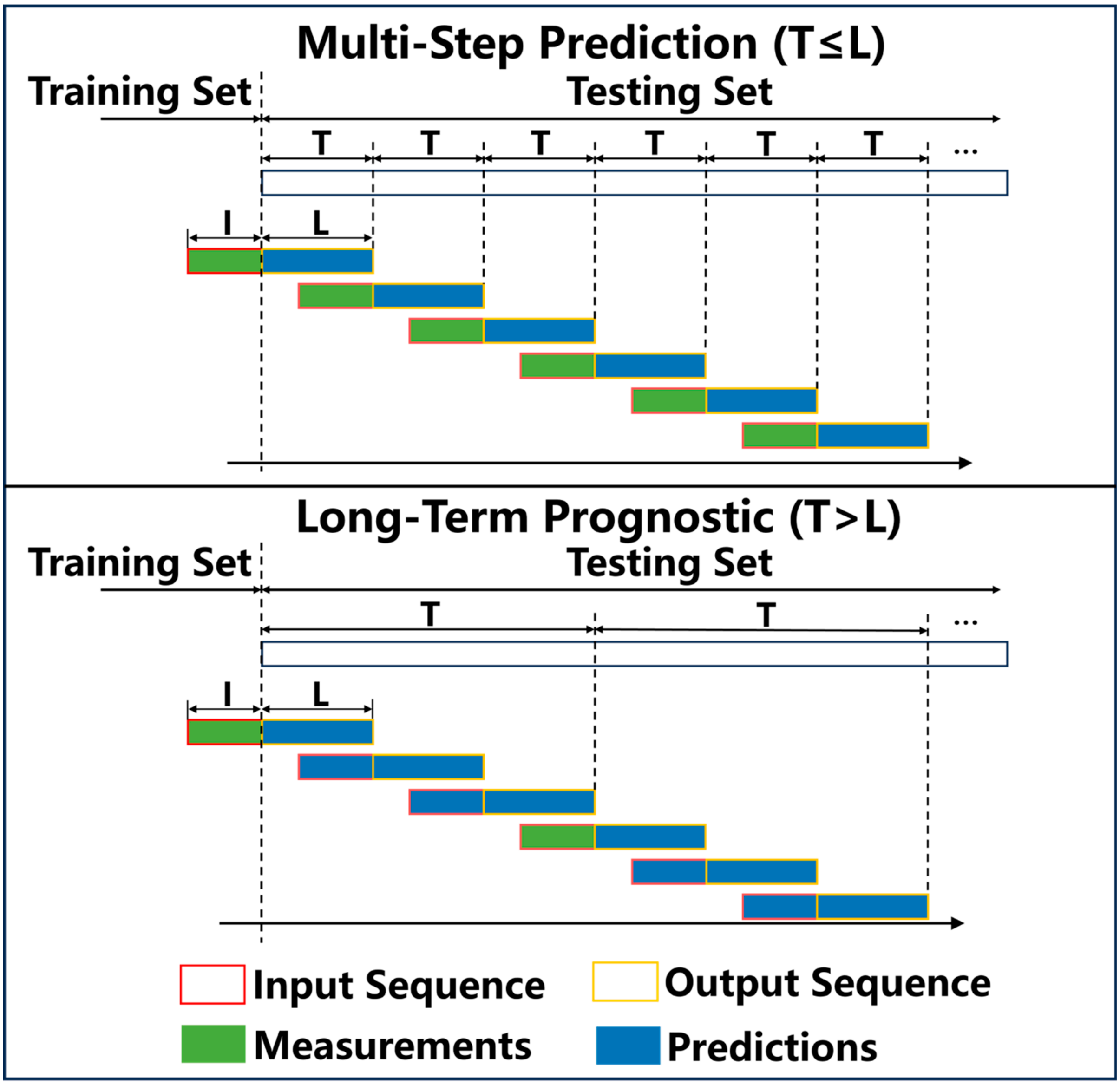


Figure S4. Difference between multi step prediction and long term prediction.

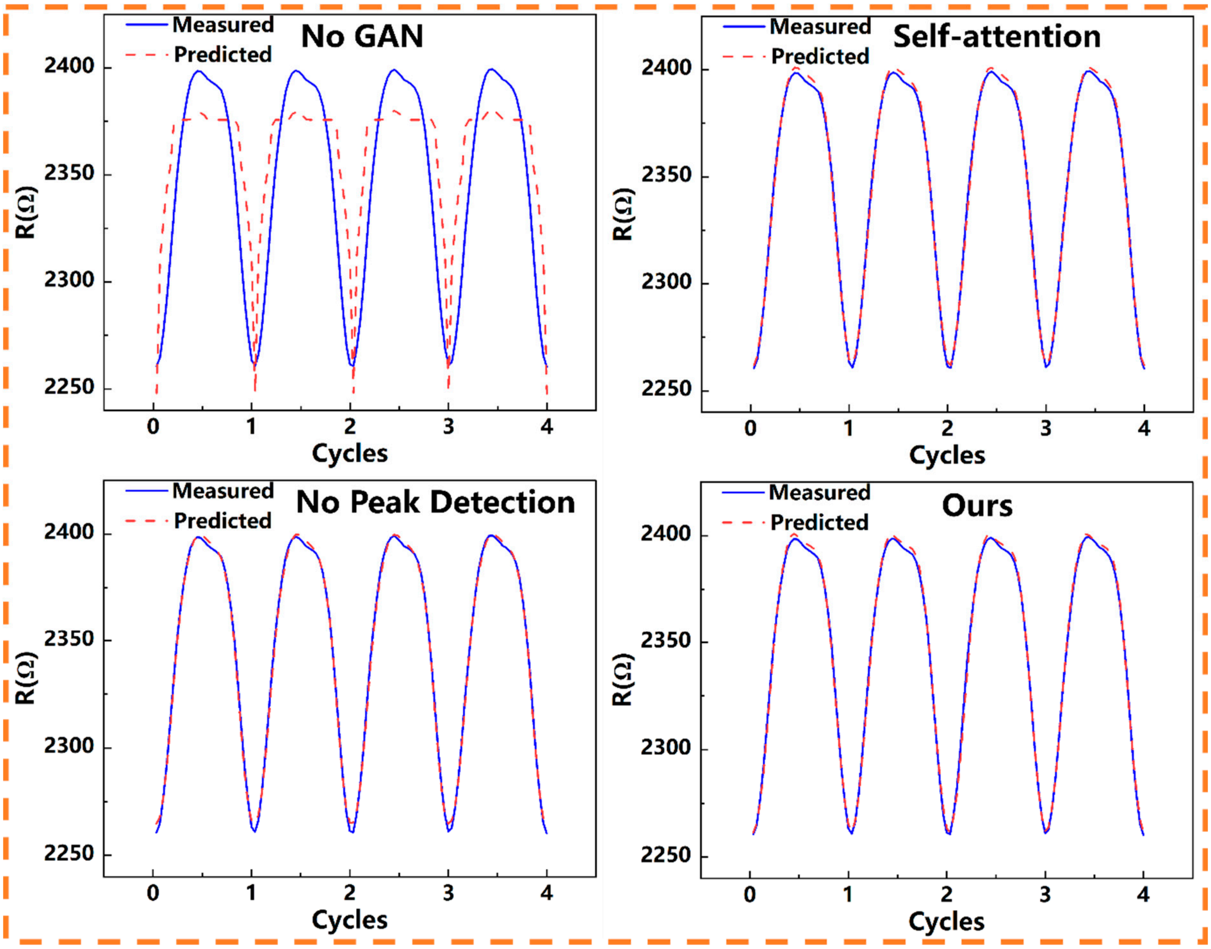


Figure S5. Comparison of prediction results from four models under 5% strain (only prediction data for the first four cycles are shown).

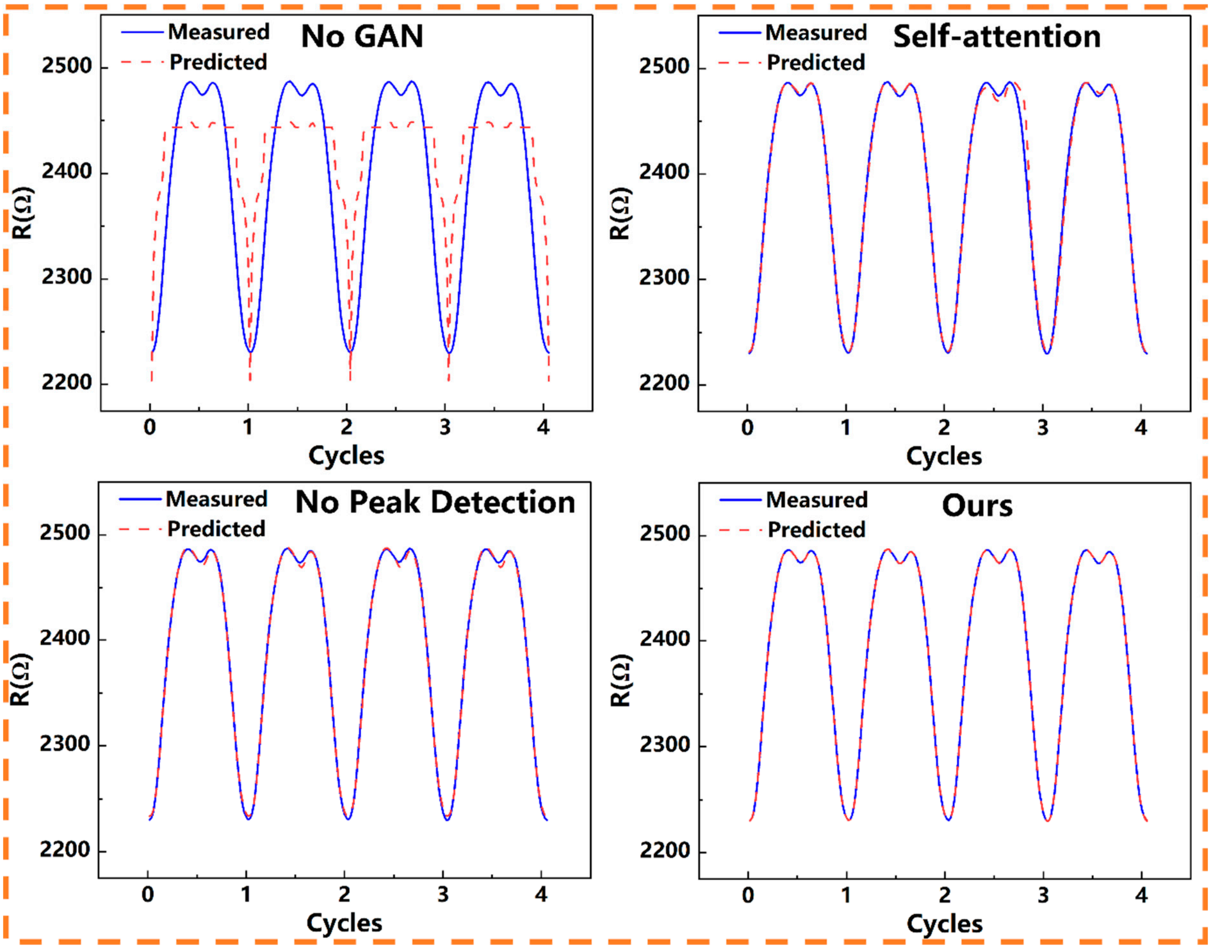


Figure S6. Comparison of prediction results from four models under 10% strain.

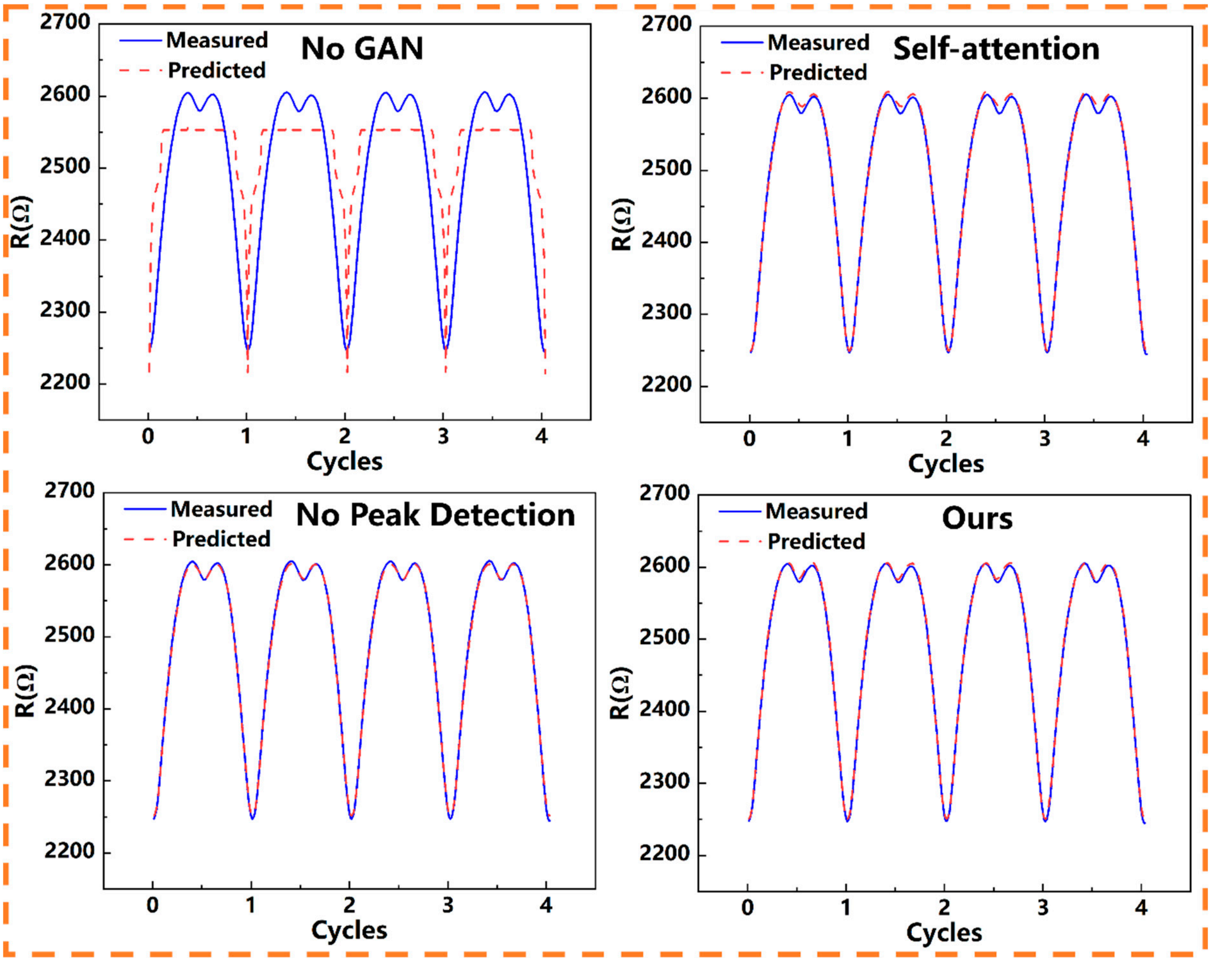


Figure S7. Comparison of prediction results from four models under 15% strain.

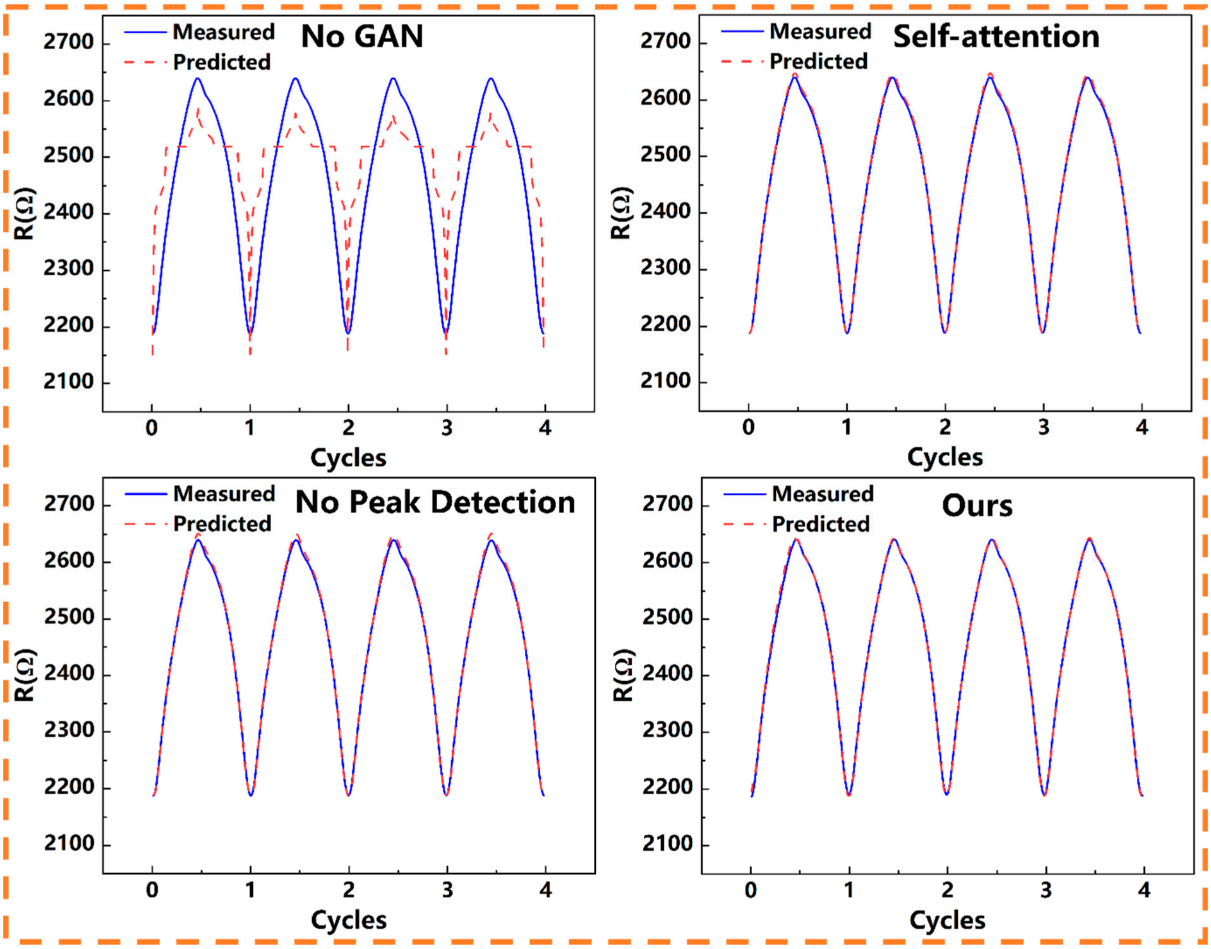

Figure S8. Comparison of prediction results from four models under 20% strain.

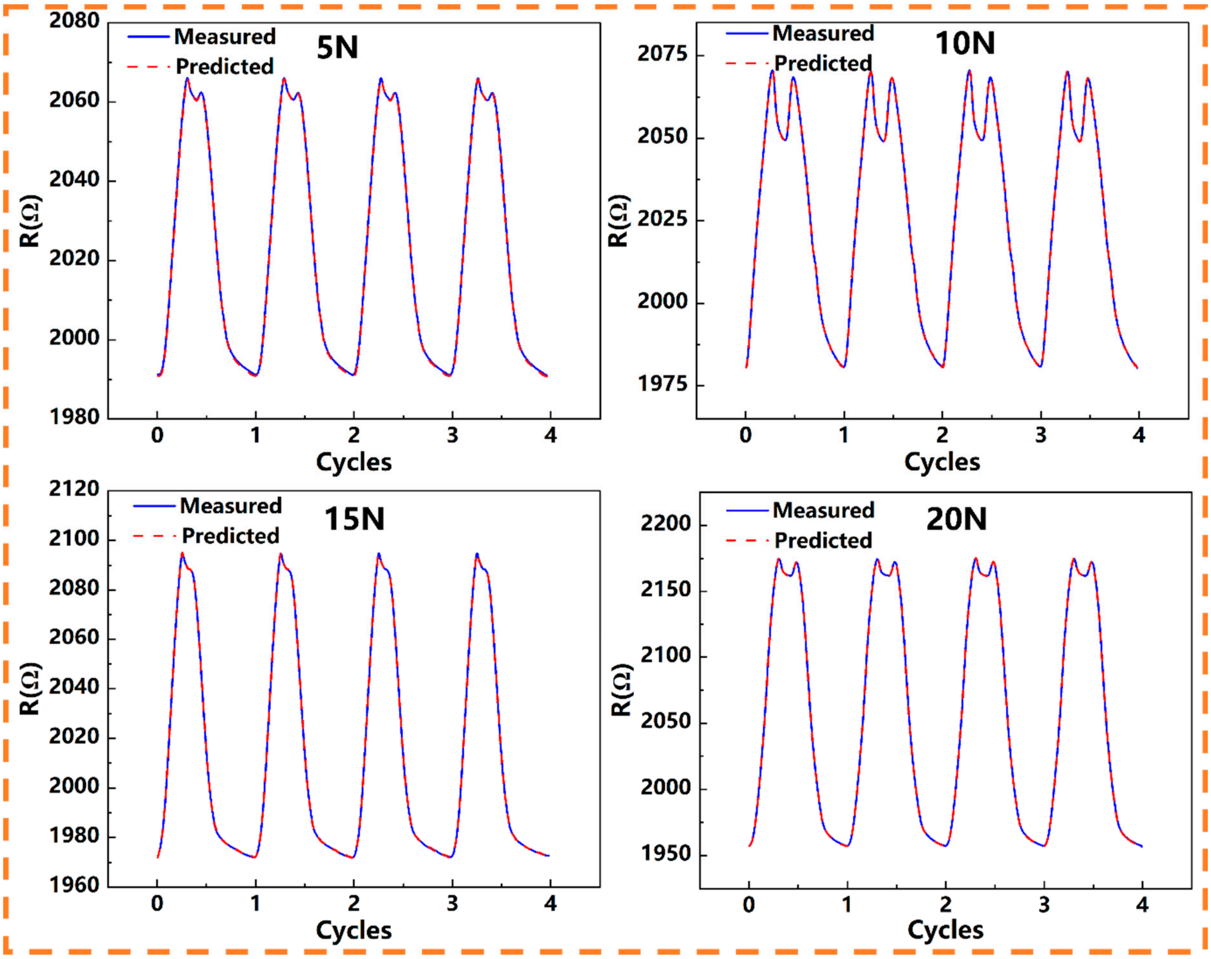


Figure S9. Comparison of prediction results under pressures of 5N, 10N, 15N and 20N.

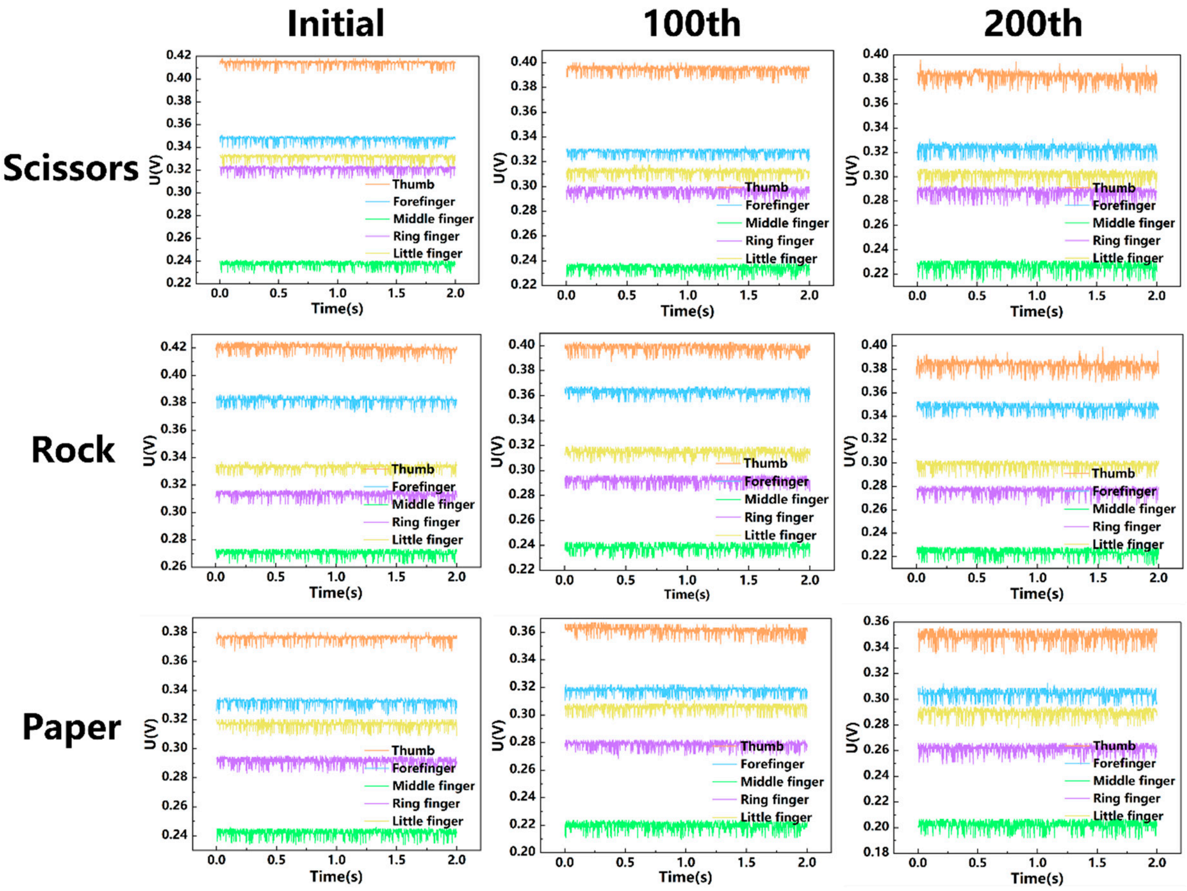


Figure S10. Voltage variations of the five sensors for different gestures in the gesture recognition task.

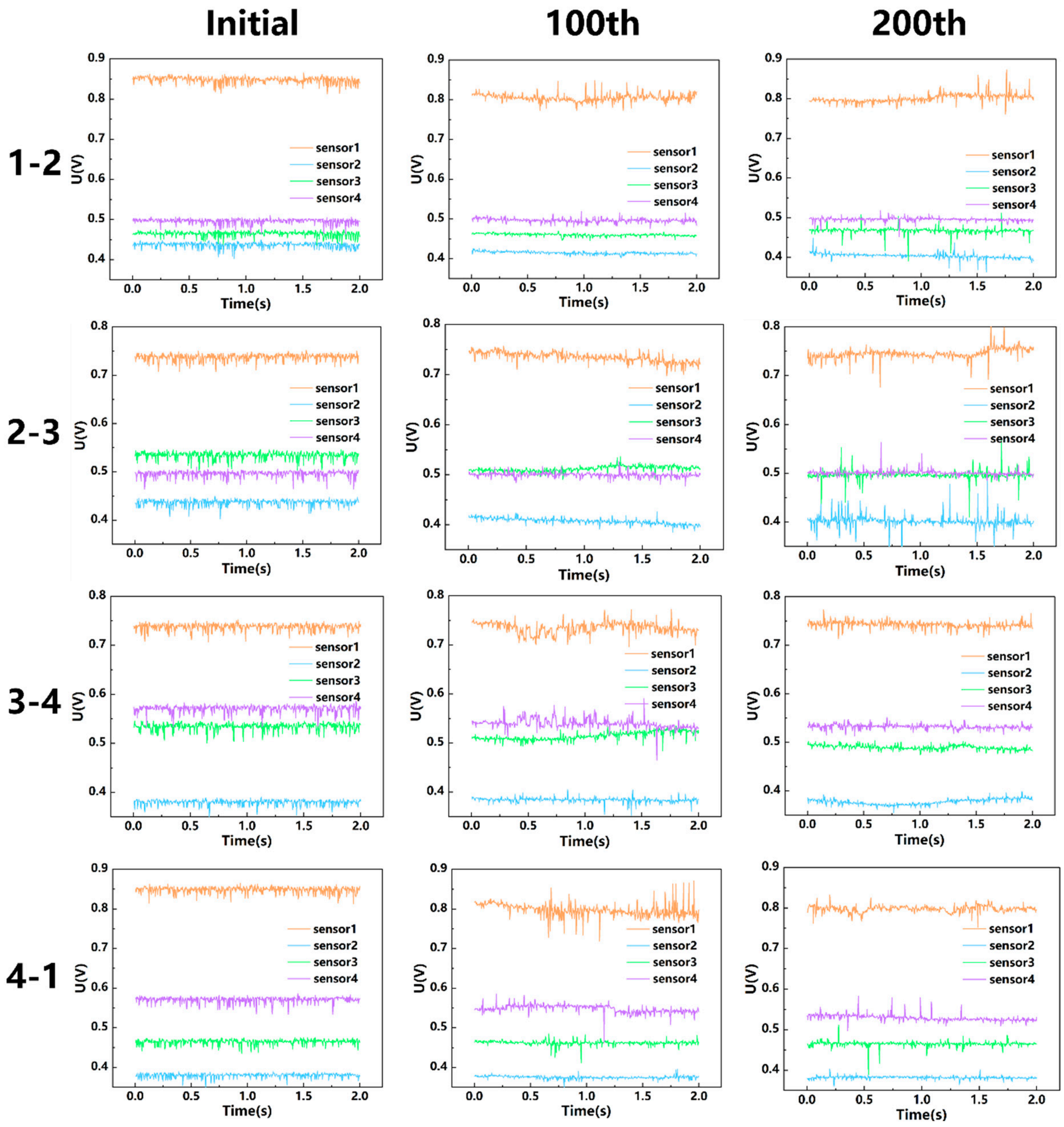


Figure S11. Voltage variations of the four sensors under tensile loads for different regions in the region recognition task.

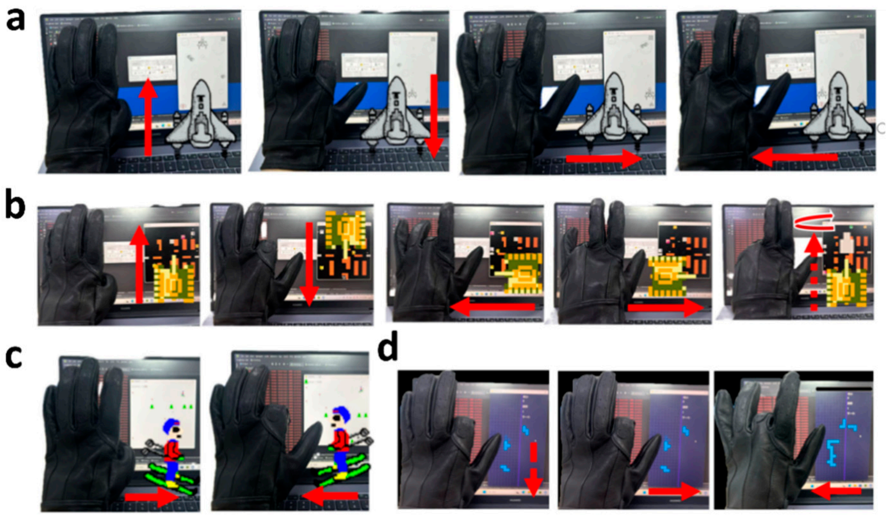


Figure S12. Smart glove applied to game control. (a) gestures to move a small airplane in each direction, (b) gestures to move a small tank in each direction and to fire, (c) gestures to move a skier, (d) Tetris control gestures.

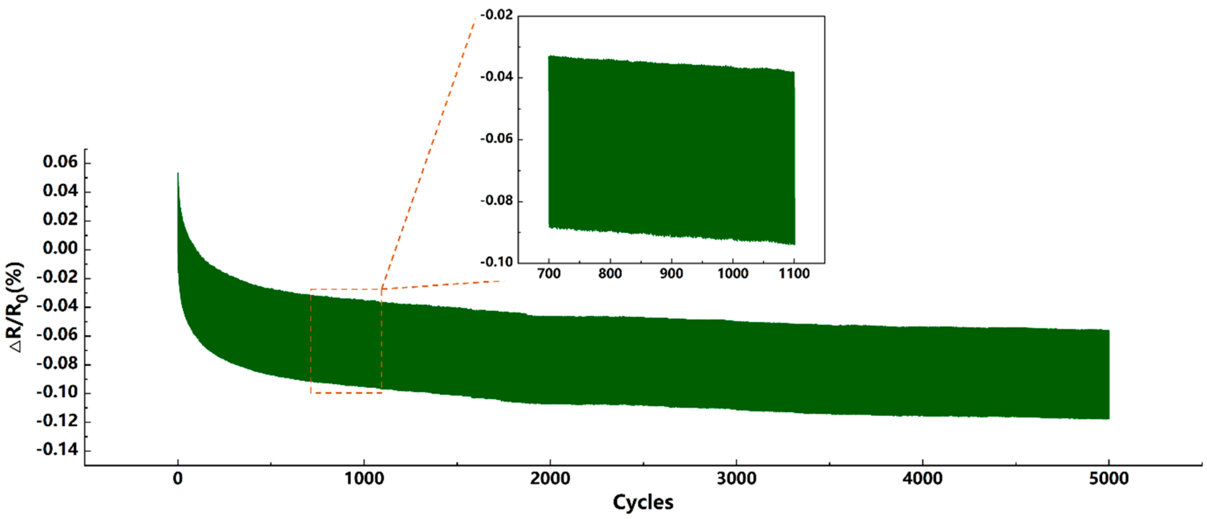


Figure S13. To ensure data representativeness and comparability the sampling begins after 700 stretching cycles and the subsequent 400 cycles are used as the experimental dataset with the final 200 cycles reserved for prediction.

Table S1. Hyperparameters of the proposed framework.

| Parameter | Values | Parameter | Values |
|---|---|---|---|
| Encoder | 2 | Optimizer | AdamW |
| Decoder | 0 | Head Number | 4 |
| Initial | 0.000015 | Feed-forward Dim | 1024 |
| Dropout | 0.1-0.4 | Feature Encoder Dim | 256 |
| Regularization | 0.000015 | Sliding Window Length | 64 |
| Test data percentage | 10% | Train data percentage | 75% |
